# Level of Detection (LOD_50_) of *Campylobacter* Is Strongly Dependent on Strain, Enrichment Broth, and Food Matrix

**DOI:** 10.3389/fmicb.2022.834568

**Published:** 2022-04-27

**Authors:** Wilma C. Hazeleger, Wilma F. Jacobs-Reitsma, Heidy M. W. Den Besten

**Affiliations:** ^1^Food Microbiology, Wageningen University & Research, Wageningen, Netherlands; ^2^Laboratory Food Microbiology, National Institute for Public Health and the Environment (RIVM), Bilthoven, Netherlands

**Keywords:** *Campylobacter jejuni*, *Campylobacter coli*, ESBL-producing *E. coli*, probability of detection, competitors, ISO 10272-1:2017

## Abstract

The detection of thermotolerant *Campylobacter* in food may be difficult due to the growth of extended-spectrum β-lactamase (ESBL)-producing *Enterobacteriaceae* during enrichment, resulting in false-negative samples. Therefore, the ISO protocol (ISO 10272-1:2017) suggests that, next to Bolton broth (BB), Preston broth (PB) is used as an enrichment broth to inhibit competitive flora in samples with suspected high levels of background microorganisms, such as ESBL-producing bacteria. However, the application of the strains used for validation of this ISO was not clearly characterized. This study examined the LOD_50_ (level of detection, the concentration where the probability of detection is 50%) of the validation strains (three *C. jejuni* and two *C. coli* strains) in BB and PB using different food matrices, namely, raw milk, chicken skin, frozen minced meat, and frozen spinach. The LOD_50_ was calculated by inoculating multiple portions with at least two inoculum levels. For each reproduction, eight test portions were used for each inoculum level and the test portion size was 10 g (chicken skin, frozen minced meat, and frozen spinach) or 10 mL (raw milk). Furthermore, the effect of artificially inoculated ESBL-producing *E. coli* on the LOD_50_ was examined to mimic the presence of ESBL-producing background microorganisms in the food matrices, namely, raw milk and chicken skin. In BB, the LOD_50_ of all strains tested in raw milk, chicken skin, and frozen spinach was rather low (0.4–37 CFU/test portion), while the LOD_50_ in frozen minced meat was higher and much more variable (1–1,500 CFU/test portion), depending on the strain. Generally, enrichment in PB resulted in higher LOD_50_ than in BB, especially for *C. coli*. Co-inoculation with ESBL-producing *E. coli* increased the LOD_50_ in BB, while PB successfully inhibited the growth of this competitive microorganism. In conclusion, food matrix and enrichment broth may have a large influence on the LOD_50_ of different *Campylobacter* strains. Therefore, it is not possible to give an unequivocal advice on when to use which enrichment broth, and this advocates the use of both methods in case of doubt. Furthermore, this study indicates specific strains that would be a good choice to use for *Campylobacter* method verification as described in ISO 16140-3:2021.

## Introduction

Thermotolerant *Campylobacter* is a major cause of human gastrointestinal infections where most cases are linked to broiler meat and raw milk (Anon., 2021a). Since the levels of this pathogen in food are generally low, and *Campylobacter* may be sublethally damaged in food matrices, an enrichment procedure is advised for the detection of this microorganism (Rogol et al., 1985; Kim et al., 2009). Several studies have been carried out to examine *Campylobacter* detection protocols showing differences in detection rates depending on the type of medium and antibiotic use (Ugarte-Ruiz et al., 2012; Jo et al., 2017). Within the International Organization for Standardization (ISO), teams of international experts drafted standards, and also a horizontal method for the detection of *Campylobacter* spp. was published (EN ISO 10272-1:2017). This standard was last revised in 2017 (Anon., 2017) and adapted to overcome the emerging problem of the presence of extended-spectrum β-lactamase (ESBL)-producing *Enterobacteriaceae* in foods (Geser et al., 2012; Reich et al., 2016). These bacteria are resistant to commonly used antibiotics in the detection media, such as cefoperazone, and also grow faster than *Campylobacter* and therefore lower the detection chance of this pathogen while being present (Jasson et al., 2009; Hazeleger et al., 2016).

The revised ISO standard defines two different enrichment procedures, using Bolton broth (BB) for the detection of low numbers of the pathogen which might be sublethally damaged and a low level of background microflora and Preston broth (PB), which is more selective, to inhibit competitive microorganisms in samples with a suspected high level of background microflora, that is, ESBL-producing microorganisms. The procedures were validated in an interlaboratory study (ILS) using frozen spinach and frozen minced meat in the BB procedure. Raw cow's milk and chicken skin were samples with expected low numbers of *Campylobacter* and a high level of background microorganisms and were validated in the PB procedure (Biesta-Peters et al., 2019). The validation resulted in a wide range of LOD_50_ (level of detection, concentration where the probability of detection is 50%), from 0.84 to 57 CFU in test portions of 10 g (Biesta-Peters et al., 2019). This large variation may be a result of the fact that the food matrices were tested using a different *Campylobacter* strain for each combination of food matrix and concomitant enrichment procedure. Although the ISO 10272-1 already indicates that the values from the ILS may not be applicable to food types or strains other than reported, applicable LOD_50_ data became even more important after the publication of ISO standard 16140-3:2021 (Anon., 2021b) for the verification of reference methods. This ISO standard describes the verification of methods in user laboratories (e.g., routine laboratories of commercial laboratories and reference laboratories of governmental agencies). This includes that their within-laboratory-determined eLOD_50_ (estimated LOD_50_) values must be evaluated against the LOD_50_ data from the ILSs of reference methods, for example, ISO 10272-1:2017.

Therefore, the aim of this study was to do a quantitative study based on the most recent ISO10272-1:2017, following up on a validation study carried out by an interlaboratory study (ILS, Biesta-Peters et al., 2019) that determined the LOD_50_ for only one strain per matrix. The current study examined the LOD_50_ of the validation strains used in the ILS of ISO 10272-1:2017 (three *C. jejuni* and two *C. coli* strains) in BB and PB using the same food matrices but in all possible combinations to determine the effect of food matrix, *Campylobacter* strain, and enrichment procedure on the probability of detection. The broiler cecal matrix was not tested in this study, since enrichment procedures are not recommended for primary production stage samples (Anon., 2017); however, the *C. jejuni* strain used for this non-food matrix in the ILS was included. Furthermore, food matrices, namely, raw milk and chicken skin, being products from animals that have been reported to contain ESBL-producing microorganisms (Geser et al., 2012), were artificially inoculated with ESBL-producing *E. coli* to mimic the presence of ESBL-producing background flora, and the effect on the LOD_50_ of *Campylobacter* was determined.

## Materials and Methods

### Strains and Culture Conditions

*Campylobacter* reference materials (Table 1) were obtained from Biosisto (Assen, Netherlands) and kept at −80°C until further use. To check the concentration, the strains were thawed, diluted in peptone physiological salt (PPS) (0.1% peptone, 0.9% NaCl; Tritium P100.25.0009, Eindhoven, Netherlands), and plated onto Columbia Agar Base (CAB) plates (Oxoid CM0331) supplemented with 5% lysed, defibrinated horse blood (Oxoid SR0050), which were then incubated for 2 days and counted to calculate the log colony-forming units per mL (log CFU/mL). The thawed reference material was kept at 4°C until the start of the experiments on Day 3. Checking of the concentration of cells in the reference material was required since, in time, different vials of the reference materials did not reliably contain the exact same levels of *Campylobacter*. The calculated concentrations were used to decide which volumes should be added to the food matrix in enrichment broth to obtain the desired two inoculation levels (determined in pre-tests; the higher level is usually 10 times higher than the lower level). It was intended to obtain a fractional recovery (preferably about 50% of the test portions to be found positive and 50% to be found negative) in at least one of the inoculation levels, to be able to calculate the LOD_50_. In the end, for each experiment, at least two inoculum levels were used, and per inoculum level, eight test portions were examined as was the case in the interlaboratory study (Biesta-Peters et al., 2019).

**Table 1:** *Campylobacter* reference strains and comparison of log LOD_50_ data calculated from the interlaboratory study (ILS) as published in ISO 10272-1:2017 (Biesta-Peters et al., 2019) with data from the current study, using Preston broth (PB) or Bolton broth (BB).

**Strain code**	**Species**	**Culture strain numbers**	**ILS matrix**	**ILS enrichment procedure**	**LOD_**50**_ in log CFU/test portion, (ILS)**	**LOD_**50**_ in log CFU/test portion (current study)**
A	*C. jejuni*	DSM 24306	Broiler cecal material	na*	0.8	nd**
B	*C. jejuni*	WDCM 00156 (ATCC 29428)	Raw milk	PB	1.8	3.7
C	*C. coli*	WDCM 00072 (ATCC 33559)	Frozen minced meat	BB	0.3	1.7
D	*C. jejuni*	WDCM 00005 (ATCC 33291)	Frozen spinach	BB	−0.1	0.3
E	*C. coli*	WDCM 00004 (ATCC 43478)	Chicken skin	PB	1.2	1.6

* *: not applicable, no enrichment procedure, only direct plating*.

nd**: not done, only food samples were examined in this study.

*Test portions were 10 g or 10 mL*.

Just before inoculation of the food matrix and mixing with the enrichment broth, the reference materials were again diluted in PPS and this working culture was plated onto CAB to check the *Campylobacter* concentration as described above to confirm the stability of the reference materials during storage at 4°C. Finally, the enrichment mixture was inoculated with the working culture, depending on the strain, enrichment broth, and food matrix. All *Campylobacter* incubations were carried out for 1–2 days at 41.5°C under microaerobic conditions, achieved by flushing anaerobic jars with 10% CO_2_, 10% H_2_, and 80% N_2_ using an Anoxomat cultivation system (I&L Biosystems, Waalwijk, Netherlands).

*Escherichia coli* strains ESBL 2 and ESBL 3 were previously isolated from raw chicken liver and chicken strips, respectively (National Institute for Public Health and the Environment). Overnight cultures in Brain Heart Infusion broth (BHI, BD 237500) were supplemented with 30% v/v glycerol in freezer vials and stored at −80°C. Before further use, strains were inoculated from the freezer vials onto a BHIA plate (BHI supplemented with 1.5% bacteriological agar no. 1, Oxoid LP0011) and incubated at 37°C for 24 h. To obtain a fresh overnight culture, one colony from the BHIA plate was inoculated in 10 mL BHI and incubated overnight at 37°C. The concentrations of the overnight cultures were determined by plating decimal dilutions in PPS onto BHIA plates. The overnight culture was kept at 4°C for 3 days until the start of the experiments, and then, the concentration was checked again as described above. ESBL-producing *E. coli* was added to the enrichment broth at a level comparable to the higher level of added *Campylobacter* which was usually between 10 and 100 CFU/test portion. This is in the same order of magnitude as reported for ESBL-containing bacteria on chicken neck skin (1–3 log CFU/g; Reich et al., 2016).

### Food Matrices

Chicken skin was kindly provided by Plukon, Wezep, Netherlands. Minced meat (beef/pork) and some additional batches of chicken skin material were obtained from a local supermarket. Ten grams of all meat and chicken skin test portions was distributed to separate stomacher bags and stored at −20°C until use. Chopped frozen spinach was also obtained from a local supermarket and kept frozen at −20°C until use. Raw cow's milk was obtained from a local dairy farm, aliquoted to 10 mL portions in 15-mL sterile Greiner tubes, and stored at −20°C until use.

Before frozen storage, food matrices were tested for the presence of *Campylobacter* [plating on RAPID' *Campylobacter* agar (RCA), Bio-Rad 3564295+3564296] and incubated at 41.5°C for 2 days and/or detection using the ISO 10272-1:2017 procedures (described below) and *Campylobacter* was not detected in 10 g or 10 mL of food matrix. The foods were further examined for ESBL-producing microorganisms using Brilliance^TM^ ESBL agar (Thermo Scientific^TM^ P05302), incubated for 1–2 days at 37°C, and total aerobic mesophilic counts using plate count agar (Oxoid CM0325), incubated for 3 days at 30°C. Usually, the ESBL-producing microorganism levels were low (<1 log CFU/g) or below the detection limit (1 log CFU/g for the solid samples or 0 log CFU/mL raw milk), and if present, they did not grow on mCCDA. In the work with adding ESBL-producing *E. coli*, however, the batch of chicken skin was *Campylobacter* positive; therefore, gamma irradiation (10 kGy, Steris, Ede, Netherlands) was used to obtain *Campylobacter*-free chicken skin samples.

### Experimental Procedure for the Detection of *Campylobacter* in Food Matrices

To determine the LOD_50_, test portions of 10 g or 10 mL of thawed food matrix sample in stomacher bags were supplemented with 90 mL of enrichment medium Bolton broth [BB, Oxoid CM0983 including SR0208E modified Bolton selective supplement and 5% lysed, defibrinated horse blood (Oxoid SR0050)] or Preston broth [PB, Oxoid CM0067 Nutrient broth no. 2 including SR0204E Preston selective supplement and 5% lysed, defibrinated horse blood (Oxoid SR0050)] and then inoculated with the working culture of *Campylobacter*. For each experiment, at least two inoculum levels were used as described above to artificially inoculate the samples. Furthermore, two blank test portions (i.e., food sample mixed with enrichment medium) without the addition of *Campylobacter* were included in each reproduction, resulting in 18 enrichment test portions per strain per matrix for each reproduction.

For detection, the enrichment part of the ISO 10272-1:2017 procedure was followed, with the omission of the BB pre-enrichment step at 37°C, because this temperature shift did not result in enhanced outgrowth of cold-stressed or non-stressed cells (Hazeleger et al., 2016). In short, BB enrichments were incubated for 48 h at 41.5°C and then streaked using a 10-μL loop onto modified charcoal cefoperazone deoxycholate agar (mCCDA, Oxoid CM0739 + SR0155E) and RCA. Plates were incubated for 48 h at 41.5°C after which suspect colonies were streaked onto CAB and incubated for 24–48 h at 41.5°C. Since the recognition of suspect colonies on mCCDA was often difficult, only colonies from RCA were used for confirmation. Colonies were checked under the microscope, and when the typical motility and spiral shape were observed, the sample was scored as positive. In case of doubt, colonies were tested with a latex confirmation assay (Microscreen, Neogen M46). The PB enrichments were incubated for 24 h at 41.5°C, and 10 μL was streaked onto mCCDA, which was incubated for 48 h at 41.5°C. Suspect colonies were then streaked onto CAB, incubated for 24–48 h at 41.5°C, and further confirmed as described above.

All four types of food test portions were inoculated with the five *Campylobacter* strains separately and tested using both enrichment procedures BB and PB in at least three independent biological reproductions. All *Campylobacter* incubations were carried out under microaerobic conditions, achieved by flushing anaerobic jars with 10% CO_2_, 10% H_2_, and 80% N_2_ using an Anoxomat cultivation system (I&L Biosystems, Waalwijk, Netherlands).

To determine the effect of ESBL-producing bacteria on the LOD_50_, *E. coli* ESBL 2 was added as co-inoculation with *Campylobacter* in the enrichment procedures with raw milk and *E. coli* ESBL 2, and ESBL 3 were added in the presence of raw chicken skin in all cases to levels of 10–100 CFU/test portion. When *E. coli* ESBL 2 or ESBL 3 was included, 10 μL of the enrichment broths was also streaked onto Brilliance^TM^ ESBL agar, incubated at 37°C for 24 h, to determine the growth or survival of the inoculated ESBL-producing *E. coli*.

### Analysis of Data

For each reproduction, the number of positive test portions per inoculation level was counted and the LOD_50_ (in CFU/test portion) was calculated using the PODLOD_ver9_test.xls program, freely available at https://www.wiwiss.fu-berlin.de/fachbereich/vwl/iso/ehemalige/wilrich/index.html (Wilrich and Wilrich, 2009). This article describes the calculation of the probability of detection, which is determined by the probability of obtaining a positive sample and the contamination of the test material, which is quantitatively expressed as the number of CFUs per unit of weight or volume. The level of detection of 50% (LOD_50_) is the contamination that gives a positive detection outcome with a 50% chance or in 50% of the samples in the current case (Wilrich and Wilrich, 2009). The LOD_50_ can be calculated by inoculating multiple test material portions with at least two inoculum levels. In this study, eight test material portions were used for each inoculum level and the test portion size was 10 g (chicken skin, frozen minced meat, and frozen spinach) or 10 mL (raw milk) to align with the interlaboratory study (Biesta-Peters et al., 2019). A higher LOD_50_ means that a higher level of *Campylobacter* is needed to detect a positive sample in 50% of the cases. Therefore, the higher the LOD_50_, the more difficult it is to detect *Campylobacter*. Only those reproductions were included that obtained a fractional recovery in either of the two inoculation levels. Since the LOD_50_ data resulted in a wide range of magnitude, the values were expressed as log CFU/test portion. If any of the blanks were positive, the food matrix was considered to be naturally contaminated after all, and that reproduction was excluded. The averages and standard deviations of different reproductions were calculated, and Student's *t*-tests were performed to determine the significance of differences (*p* < 0.05) between different food matrices, strains, and conditions. All calculations were done in Excel.

To be able to properly compare the data from this study to the ILS data, log LOD values were calculated for the ILS data as published by Biesta-Peters et al. (2019) as well (Table 1).

## Results

### LOD_50_ of *Campylobacter* in Bolton Broth

**Figure 1:**
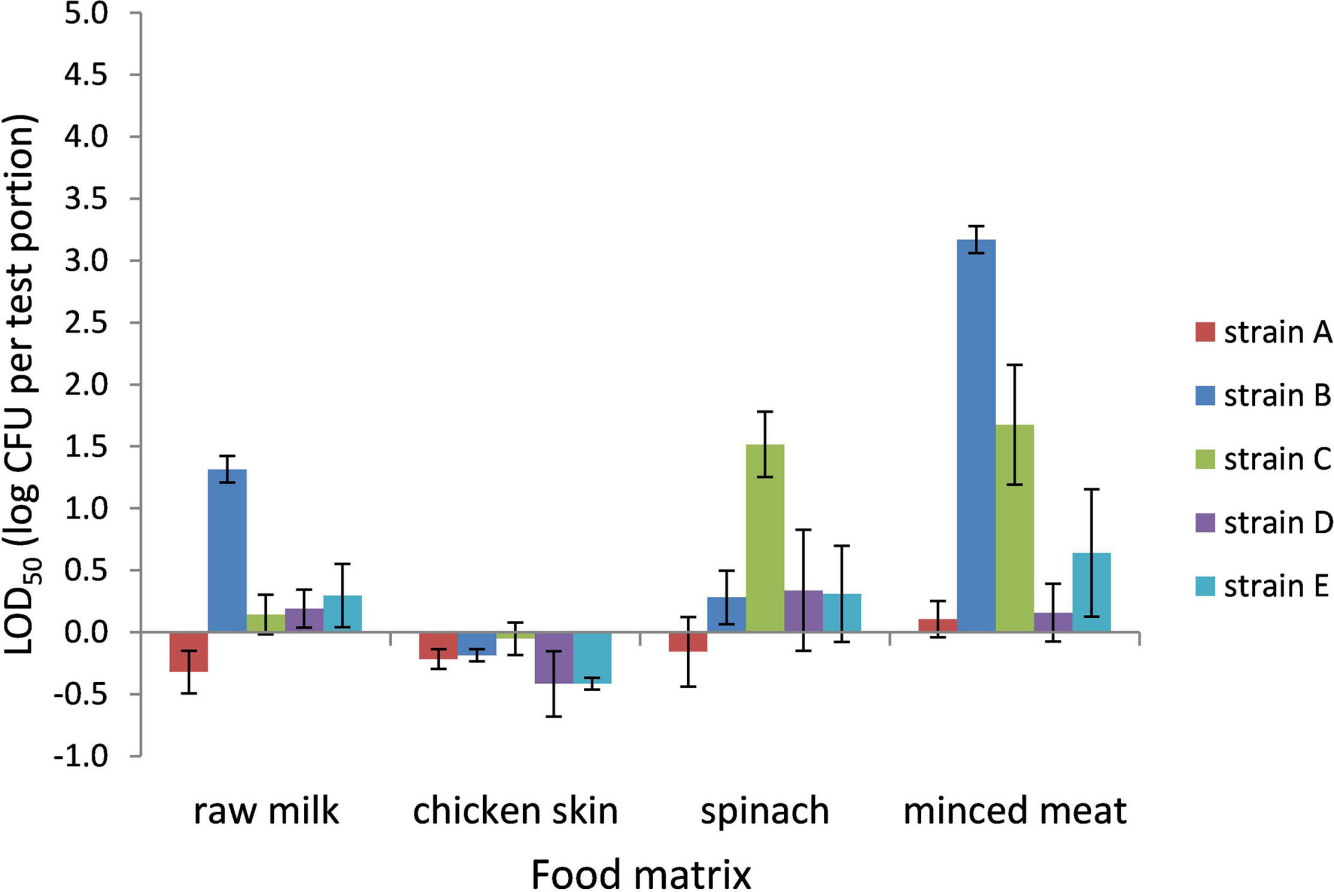
LOD_50_ of *C. jejuni* strains A, B, and D and *C. coli* strains C and E. Enrichment in Bolton broth, including food matrices, namely, raw milk (*n* = 3), chicken skin (*n* = 2–5), frozen spinach (*n* = 3), and frozen minced meat (*n* = 3–4). Error bars indicate the standard deviation.

The LOD_50_ for *Campylobacter* in BB ranged from 0.4 CFU per test portion in chicken skin to 1,500 CFU per test portion in frozen minced meat (−0.4 log CFU per test portion to 3.2 log CFU per test portion, respectively) and was dependent on the food matrix and strain (Figure 1). In general, the LOD_50_ in chicken skin was low for all strains (<1 CFU per test portion). Also, in raw milk and frozen spinach, a relatively low LOD_50_ was observed as 0.5 (strain A) to 21 CFU (strain B) per test portion and 0.8 CFU (strain A) to 37 CFU (strain C) per test portion, respectively. In frozen minced meat, the five strains showed the highest variability in LOD_50_, with significantly higher levels for strains B and C (*p* < 0.05). Strains A and D demonstrated a low LOD_50_ compared with the other strains, and differences between the food matrices were limited.

### LOD_50_ of *Campylobacter* in Preston Broth

**Figure 2:**
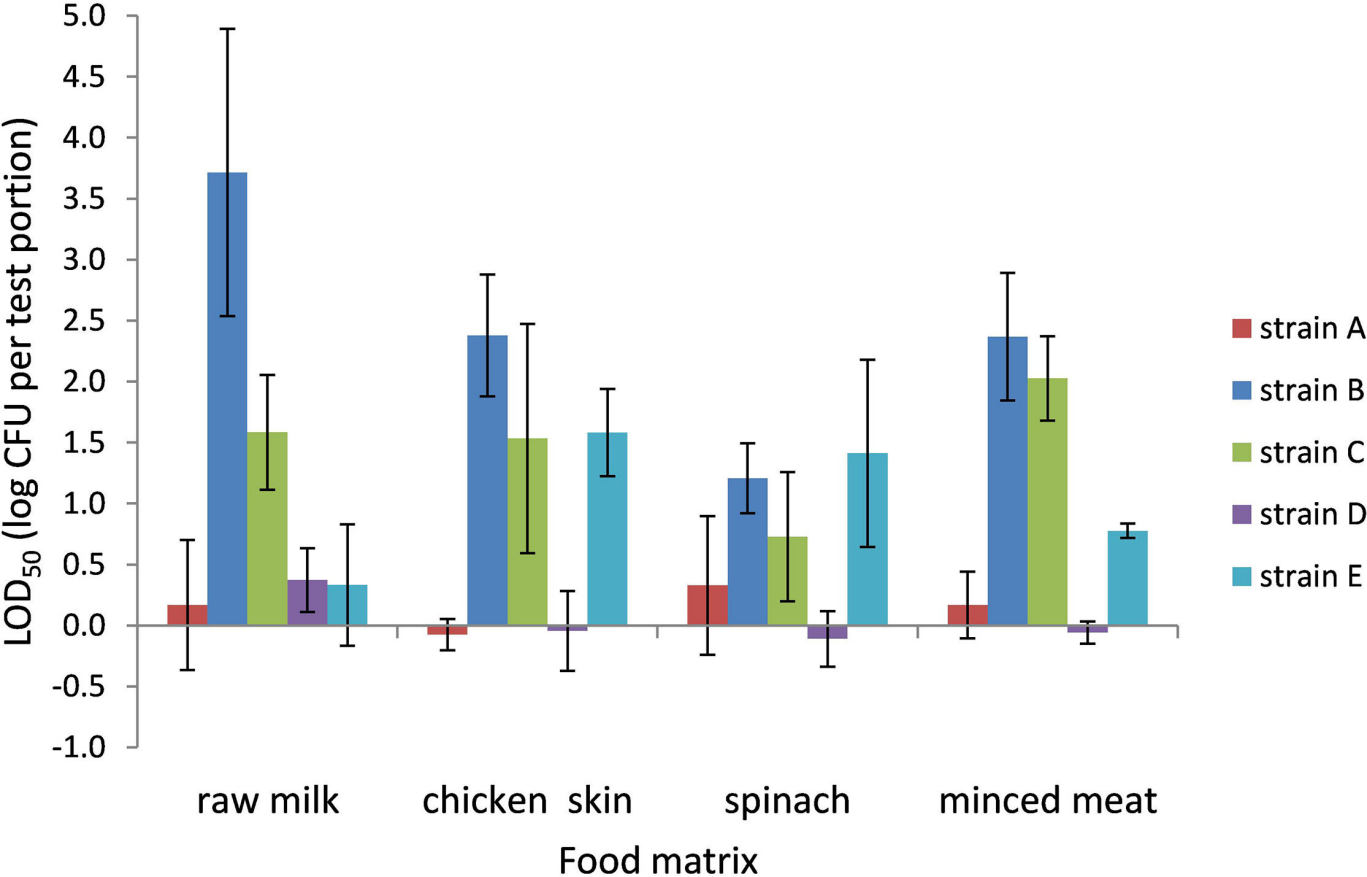
LOD_50_ of *C. jejuni* strains A, B, and D and *C. coli* strains C and E. Enrichment in Preston broth, including food matrices, namely, raw milk (*n* = 3), chicken skin (*n* = 3–4), frozen spinach (*n* = 3–4), and frozen minced meat (*n* = 2–3). Error bars indicate the standard deviation.

The LOD_50_ for *Campylobacter* in PB ranged from 0.9 CFU per test portion in chicken skin to 8,900 CFU per test portion in raw milk (−0.05 to 3.7 log CFU per test portion, respectively) and was dependent on the food matrix and strain (Figure 2). All food matrices showed high variability in LOD_50_ depending on the strain; especially strains B and C, and to a lesser extent also strain E, showed higher LOD_50_ compared with strains A and D.

When compared to the data in BB, the LOD_50_ in PB was often higher than in BB, but only significant (*p* < 0.05) for strain B (raw milk, chicken skin, and frozen spinach), strain C (raw milk), and strain E (chicken skin).

### LOD_50_ of *Campylobacter* in Raw Milk and Chicken Skin With Artificial Inoculation of ESBL *E. coli*

**Figure 3:**
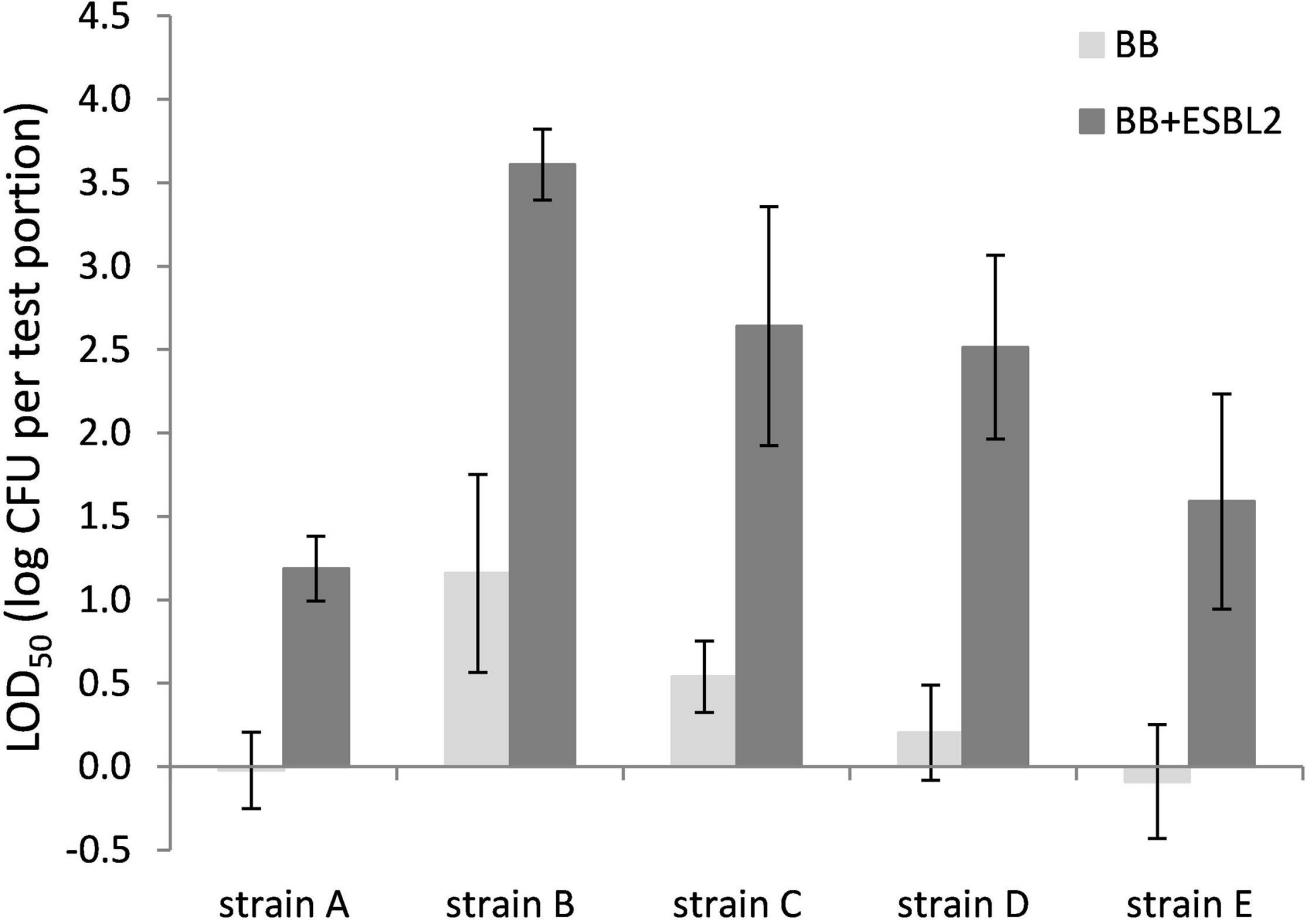
LOD_50_ of *C. jejuni* strains A, B, and D and *C. coli* strains C and E. Enrichment in Bolton Broth (*n* = 2–7) and with co-inoculation of *E. coli* ESBL 2 (*n* = 2–5) in raw milk. Error bars indicate the standard deviation.

Co-inoculation with *E. coli* ESBL 2 in BB resulted in significantly higher LOD_50_ for all *Campylobacter* strains tested in raw milk (Figure 3). In PB, however, the growth of ESBL-producing *E. coli* was successfully inhibited, resulting in similar LOD_50_ values for *Campylobacter* as shown in Figure 3, regardless of co-inoculation with *E. coli* ESBL 2 (data not shown).

**Figure 4:**
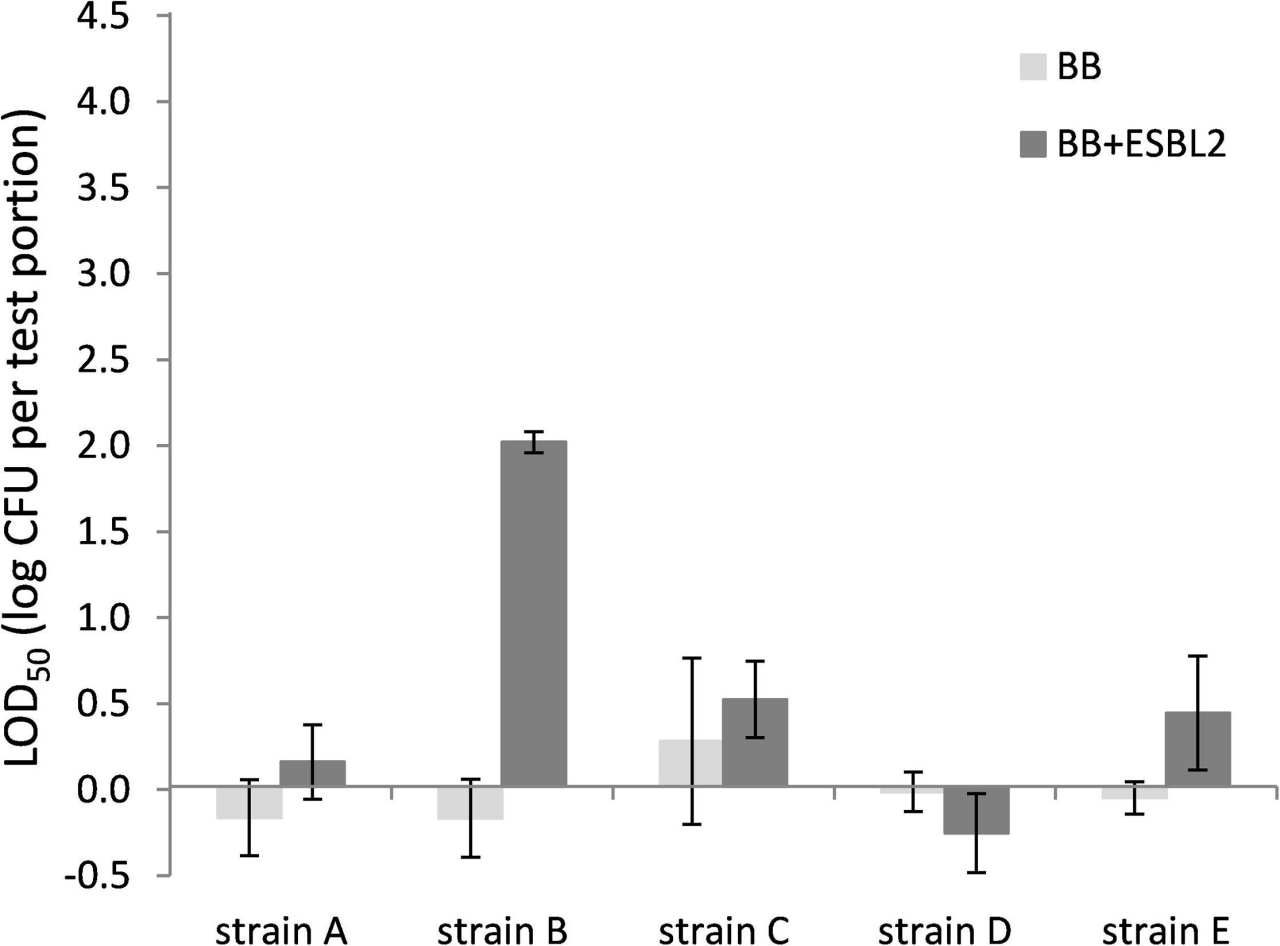
LOD_50_ of *C. jejuni* strains A, B, and D and *C. coli* strains C and E. Enrichment in Bolton broth (*n* = 2–5) and with co-inoculation of *E. coli* ESBL 2 (*n* = 2–3) in chicken skin (irradiated before use). Error bars indicate the standard deviation.

Also for chicken skin, the co-inoculation with *E. coli* ESBL 2 in BB resulted typically in higher LOD_50_, although differences were not significant for all strains (Figure 4). When strain *E. coli* ESBL 3 was included, similar trends were observed as shown in Figure 4 for *E. coli* ESBL 2 (data not shown).

When *E. coli* ESBL 2 or ESBL 3 was included in the BB enrichments, the isolation medium mCCDA was in more than 99% of the cases completely covered with ESBL-producing colonies, stressing the need for the second isolation medium as advised by ISO 10272-1:2017. In this study, RAPID' *Campylobacter* agar was used as the second plating medium which showed to be selective against *E. coli* ESBL. In the PB enrichments, *E. coli* ESBL colonies were found on <1% of the mCCDA plates, indicating that these bacteria were well-inhibited during the incubation in PB.

## Discussion

The aim of this study was to examine the effect of food matrix, strain, and enrichment broth on the probability of detection of three *C. jejuni* and two *C. coli* strains, previously used for validation of the ISO 10272-1:2017 (Anon., 2017; Biesta-Peters et al., 2019).

Comparing the LOD_50_ published from the interlaboratory study (ILS) (Table 1; Biesta-Peters et al., 2019), to the data found in this study, it can be concluded that in both studies, strain B has the highest LOD_50_ (1.8 and 3.7 log CFU/test portion, respectively) and strain D has the lowest LOD_50_ (−0.08 and 0.3 log CFU/test portion, respectively). The LOD_50_ values in the current study were higher than the ILS (Table 1), and the standard deviation was high, highlighting the high difference between replicate experiments. Differences in LOD_50_ between the ILS and this study might be explained by different procedures followed. The ILS used frozen *Campylobacter* reference materials that were transported in dry ice and defrosted on the day of the experiment (Biesta-Peters et al., 2019), and each laboratory used the same batch of materials. In this study, we performed independent biological reproductions and thawed reference materials were kept for 3 days at 4°C and only then used in the experiments. This procedure was required since, in time, different vials of the reference materials did not reliably contain the exact same levels of *Campylobacter*. As a result, the concentrations had to be tested first and then the thawed materials were kept to be certain of the exact concentration on the day of testing to inoculate the test portions at such levels as to obtain a fractional recovery for calculation of the LOD_50_. Although the *Campylobacter* concentrations did not alter significantly during storage at 4°C, still the metabolic status or stress level of the microorganism could have been influenced and may have resulted in a different recovery during enrichment, which might be dependent on the strain (Lanzl et al., 2020). However, also when the procedures were the same, comparing the LOD_50_ in BB (Figure 1), to the data of the later studies in BB (Figures 3, 4), showed that the LOD_50_ of strain C in raw milk and strain E in chicken skin were significantly higher in the later study (*p* < 0.05). Therefore, a bias due to laboratory workers, number of biological reproductions, time, batch of food matrix, or other unknown conditions could not be excluded.

The large differences in LOD_50_ between the different strains in Bolton broth did not seem to be caused by the antibiotics in the broth alone, since for all strains in chicken skin, a low LOD_50_ was obtained. Especially frozen minced meat proved to be problematic for strains B and C, while this could not be related to the levels of background microorganisms, which were similar compared with chicken skin (total aerobic mesophilic counts of 5–6 log CFU/g). Since the type of background flora was not extensively investigated, specific groups of flora could have been of influence; however, it was determined that the samples did not contain detectable levels of ESBL-producing bacteria (<1 log CFU/g). Besides the nature of the background microorganisms, also the composition of the foods may have played a role, for instance, the addition of antioxidants sodium ascorbate or sodium citrate in frozen minced meat which may inhibit *Campylobacter* as well (Juven et al., 1988; Meredith et al., 2013) or the presence of the lactoperoxidase system in raw milk which may lead to a reduction in *C. coli* and *C. jejuni* (Beumer et al., 1985; Ronacher et al., 2003). Overall, strain A performed best in all food matrices and strains D and E relatively well, whereas strains B and C resulted in comparatively high LOD_50_.

Preston broth contains polymyxin B which is a good inhibitor of ESBL-producing microorganisms (Habib et al., 2011; Hazeleger et al., 2016) and inhibits the growth of most gram-negative strains (Baylis et al., 2000; Paulsen et al., 2005), including some *C. jejuni* and especially *C. coli* strains (Goossens et al., 1986). This might explain why *C. jejuni* strain B and *C. coli* strains C and E resulted in high LOD_50_ values in the experiments with PB and agreed with the relatively high LOD_50_ found in the ILS using PB for *C. coli* strain E in chicken skin and *C. jejuni* strain B in raw milk.

Since most food matrices in this study did not contain detectable levels of ESBL-producing microorganisms (<1 log CFU/g), the effect of artificially inoculated *E. coli* ESBL was examined to mimic the presence of ESBL-producing background flora. For these experiments, the food matrices, namely, raw milk and chicken skin, were chosen since these were the matrices potentially containing high levels of background microorganisms (Dierikx et al., 2010; Biesta-Peters et al., 2019). As expected, in raw milk in Bolton broth, the LOD_50_ of all strains significantly increased upon co-inoculation with *E. coli* ESBL, which could be explained by faster growth to higher levels of *E. coli* ESBL compared with *Campylobacter* (Hazeleger et al., 2016), making the detection of *Campylobacter* complicated. When using Preston broth, the LOD_50_ was similar, irrespective of co-inoculation with *E. coli* ESBL. This observation confirms the preference for using PB over BB as a choice for the detection of *Campylobacter* in samples with possible ESBL-producing natural contaminants as described by ISO 10272-1 (Anon., 2017). With co-inoculation of *E. coli* ESBL 2 and ESBL 3 in the chicken skin matrix in BB, the LOD_50_ was also increased for strains A, B, C, and E, but this was only significant for strain B (*p* < 0.05). Unfortunately, the batch of chicken skin used for this part of the study was naturally contaminated with *Campylobacter*, which made this batch unfit for immediate use since the natural presence of unknown levels of *Campylobacter* would interfere with the LOD_50_ calculation. Therefore, the chicken skin was gamma-irradiated to obtain *Campylobacter*-free chicken skin. As a consequence, all natural background flora was also killed, making the outcome of this experiment difficult to compare to the earlier experiments with natural background, although the relatively low LOD_50_ indicates that the natural microorganisms from the earlier chicken skin samples also did not impede *Campylobacter* detection. Apparently, the detection of the pathogen in chicken skin matrix was quite efficient, which was also observed in a previous study with naturally contaminated chicken liver samples that were co-inoculated with *E. coli* ESBL (Hazeleger et al., 2016).

The high LOD_50_ (up to 8,900 CFU per test portion of 10 g), as found in some cases in this study, could be problematic, since the probability of illness was shown to be 45–70% at the relatively low dose of 10 CFU (Teunis et al., 2005) which was estimated to be dependent on age and type of food, such as liquid or solid food (Abe et al., 2021). Therefore, the current methods as described in the ISO 10272-1:2017 may not be sensitive enough to detect the relatively low levels of *Campylobacter* that may cause disease and stresses the need for improvement in the detection procedures. For example, if novel media are both selective and promote the growth of all thermotolerant campylobacters, potentially in combination with the use of molecular methods for the detection of *Campylobacter* (Ugarte-Ruiz et al., 2012; Da Silva Frasao et al., 2017).

The fact that some strains perform better than others could also be important in performance testing of (enrichment) media, which is originally described in standard ISO 11133 (Anon., 2014). The choices for test strains *C. jejuni* D (WDCM 00005) and *C. coli* E (WDCM 000004) are supported by this study, since they performed relatively well in most food matrices tested. However, strain B (WDCM 00156), which is also suggested in ISO 11133:2014 and ISO 10272-1:2017, does not seem to be a good choice as demonstrated in raw milk and especially frozen minced meat.

The indication in ISO 10272-1:2017 that the values from the interlaboratory study may not be applicable to food types or strains other than reported is strongly confirmed in this study. Simultaneously, ISO standard 16140-3:2021 (Anon., 2021b) describes the verification of methods in user laboratories and their determined eLOD_50_ (estimated LOD_50_) values must be evaluated against the LOD_50_ data from the ILSs of reference methods, for example, ISO 10272-1:2017. Therefore, applicable LOD_50_ data are important, and from this study, it can be concluded that *C. jejuni* strains A and D and *C. coli* strain E would be a good choice to use for *Campylobacter* method verification as described in ISO 16140-3:2021.

## Conclusions

In conclusion, food matrix and type of enrichment broth (Bolton or Preston) may have a large influence on the LOD_50_ of different *Campylobacter* strains. Therefore, it is not possible to give an unequivocal advice on when to use which enrichment broth, and this advocates the use of both methods in case of doubt.

Furthermore, this study indicates that *C. jejuni* DSM 24306, *C. jejuni* WDCM 00005 (ATCC 33291), and *C. coli* WDCM 00004 (ATCC 43478) would be good choices to use for *Campylobacter* method verification as described in ISO 16140-3:2021.

## Data Availability Statement

The raw data supporting the conclusions of this article will be made available by the authors, without undue reservation.

## Author Contributions

WH, WJ-R, and HD conceived the study, wrote the manuscript, interpreted the data, and approved the submitted version of the manuscript. WH and HD carried out the data analysis. All authors contributed to the article and approved the submitted version.

## Funding

This work was funded by the University as part of a regular research project.

## Conflict of Interest

The authors declare that the research was conducted in the absence of any commercial or financial relationships that could be construed as a potential conflict of interest.

## Publisher's Note

All claims expressed in this article are solely those of the authors and do not necessarily represent those of their affiliated organizations, or those of the publisher, the editors and the reviewers. Any product that may be evaluated in this article, or claim that may be made by its manufacturer, is not guaranteed or endorsed by the publisher.

## Abbreviations

BB: Bolton broth
CAB: Columbia agar base
ESBL: extended-spectrum β-lactamase
ILS, interlaboratory study (Biesta-Peters et al.: 2019)
LOD_50_, level of detection: concentration where the probability of detection is 50%
mCCDA: modified charcoal cefoperazone deoxycholate agar
PB: Preston broth
RCA: RAPID&#8217
*Campylobacter* agar.
